# Case of Facial Neuralgia

**Published:** 1871-12

**Authors:** John Mcsheehy


### ARTICLE VIII.

### *Case of Facial Neuralgia.*

BY JOHN MCSHEEHY, M. D.

D. C., aged 42, applied to me June 17th, 1871, suffering from paroxysms of severe pain, mostly of a plunging lancinating character, shooting in the course of the facial nerves and extending under the molars of the left side. He had felt no trouble in the superior maxillary of that side. The patient had applied for medical treatment four years previously in Montana Territory, and again in New York, without obtaining any relief. He had tried all known remedies without success. Visited Boston to see his sister, and called on me as before mentioned.

On making an examination, I found that three molar teeth had been extracted by advice previous to his arrival. A spongy state of the gums existed, with a slight ulceration and fœtid breath. Paroxysms of pain occurred every ten or fifteen minutes, preventing sleep. When he became overpowered by sleep he was at once awakened, and star ed up screaming with the severe pains.

A faithful use of cinchona, iodoform and ferri valerianat. caused some general improvement up to the 30th of June, at which time I commenced subcutaneous injections and also the employment of hydrate of chloral, without favorable result. The following lotion was also employed, but without avail:—

℞. Ætheris sulph., ʒ ij.;

Chloroformi,

Tincturæ opii,

Olei terebinthinæ,

Tincturæ capsici, aa ʒ iij.;

Extracti belladonnæ, ʒ i. M.

No ease was obtained from any liniment or medicinal preparation whatever. I made an examination of the teeth and gums on the 2d of July, and found what I had supposed necrosis of the alveolar process of the superior maxilla on the left side. I recommended extraction of the diseased bone, as I thought, an opinion I had formed because the apparent sequestrum was above the alveolar process. I consulted with Dr. Dan'l G. Harkins, surgeon dentist, of Tremont Street, and we concluded to extract what appeared to be the necrosed bone, but which, on extraction, proved to be a dens sapientia covered with a fleshy bulb and imbedded in the superior maxilla. The tooth had pressed on the trunk of the infra-dental nerve in its course, and had caused pain of the most excruciating character, which the patient had referred to the lower maxillary bone.

Since the wisdom tooth has been extracted he has not had a single paroxysm of pain, with the exception of one or two momentary flashes. He feels well and free from pain, and has slept sounder since than for four years previous, and intends to return to Montana Territory to resume his mining operations.—*Boston Medical and Surgical Reporter.*

---

